# Post-Extraction Bone Changes in Molars Within Personalized Implant-Prosthetic Therapy as Evaluated with Fractal Analysis of CBCT

**DOI:** 10.3390/jpm15040154

**Published:** 2025-04-16

**Authors:** Antonia Samia Khaddour, Sanda Mihaela Popescu, Mihaela Ionescu, Alex Ioan Sălan, Răzvan Eugen Ghiţă, Melania Olimpia Cojocaru, Iulia Roxana Marinescu, Marina Olimpia Amărăscu, Emma Cristina Draghici

**Affiliations:** 1 Department of Oral Rehabilitation, University of Medicine and Pharmacy of Craiova, 200349 Craiova, Romania; antoniasamia11@gmail.com (A.S.K.); razvan.ghita@umfcv.ro (R.E.G.); melania.cojocaru@umfcv.com (M.O.C.); iulia.marinescu@umfcv.com (I.R.M.); emma.draghici@umfcv.ro (E.C.D.); 2 Department of Medical Informatics and Biostatistics, University of Medicine and Pharmacy of Craiova, 200349 Craiova, Romania; 3 Department of Oral and Maxillofacial Surgery, University of Medicine and Pharmacy of Craiova, 200349 Craiova, Romania; alex.salan@umfcv.ro; 4 Department of Dental Technology, University of Medicine and Pharmacy of Craiova, 200349 Craiova, Romania; marina.amarascu@umfcv.ro

**Keywords:** personalized implant-prosthetic therapy, tooth extraction, molar, fractal analysis, CBCT, bone structure, ImageJ

## Abstract

**Background**: Implant-prosthetic therapy requires a detailed assessment of the bone structure before designing a personalized treatment plan. Tooth extraction at the molar level is followed by a series of bone changes dependent on the patient’s general condition and age and the area in which it was performed. The fractal analysis of cone beam computed tomography (CBCT) represents a way to assess the quality of post-extraction bone regeneration. The purpose of this study was to analyze the alveolar bone changes after tooth extraction using fractal analysis on CBCT images. **Methods**: This retrospective study included pre- and post-extraction CBCTs at 3 months of 60 patients who underwent 100 extractions of first and/or second molars. Fractal analysis on CBCT images was performed using ImageJ, and the data obtained from the measurements were statistically processed. A multiple regression model was used to assess factors influencing bone remodeling. **Results**: Fractal analysis performed on CBCT images showed that most patients experienced advanced bone remodeling, this being more pronounced in those from rural areas, in the vertical plane at the mandible and at the second molar. The multiple regression model showed that the factors that play an important role in predicting bone resorption are represented by age group (age above 56 years old is associated with less bone resorption), location (bone resorption is more pronounced at the mandible level), and molar (bone resorption for the second molar is higher). **Conclusions**: Post-extraction bone changes were influenced by the age of the patient and by the location of the extraction, with the maxilla and first molar having better fractal analysis values compared to the mandible and second molar. These results emphasize the importance of training implantologists in CBCT evaluation to improve personalized implant-prosthetic treatment decisions.

## 1. Introduction

Implant-prosthetic therapy is a highly personalized type of therapy, which starts from the detailed evaluation of the patient (general condition, oral health, and maxillary bone condition) to the decision to choose the type of implant and prosthetic work and their application in the oral cavity. The planning of the implant-prosthetic restoration must begin from the moment of the decision of tooth extraction so that means can be used to improve the success of the long-term treatment. These refer to extraction techniques and techniques for preserving the size of the alveolar process, as well as implant insertion and prosthetic techniques. In this entire personalized therapeutic approach, the bone evaluation stage with modern technologies, such as cone beam computed tomography (CBCT)/fractal analysis (FA), greatly help the doctor in choosing the extraction technique, the type of biomaterial used for bone preservation, as well as the type of implant used for the kind of bone in the respective area. Without a detailed analysis of all these elements, the long-term success of personalized implant prosthetic therapy cannot be ensured.

The success of the implant therapy depends to a great extent on the health of the jawbones and the peri-implant soft tissues. Alveolar bone changes inevitably occur, in various degrees and forms, after tooth extraction, making the quality of the remaining bone an important aspect to consider [1,2,3]. The highest rate of bone resorption occurs in the first 6 months after tooth extraction [4], with the height decreasing rapidly in the first 3 months [5]. In addition, bone resorption has been shown to be higher in the molar region [6,7] and especially in the maxilla [2,8]. The loss of alveolar bone volume and the concomitant decrease in mineral density were first studied in 1960 [9]. Radiological evaluation of the quantitative and qualitative alveolar bone is crucial for establishing a successful implant treatment plan. One such investigation method is the evaluation of the trabecular pattern using radiological analysis [10].

The three-dimensional imaging investigation method performed using CBCT has become an indispensable method in all branches of dentistry [11,12,13,14,15,16], as it allows for obtaining detailed and high-quality reformatted images [17,18]. In addition, this method offers multiple other advantages, such as a low radiation dose or improved resolution [11,19]. CBCT is an essential tool for establishing the implant treatment plan because, through it, we can easily identify patients at risk of low bone density [10,20].

In 1980, Mandelbrot first introduced the concept of fractal analysis [21,22,23], which can be numerically expressed as fractal dimension [21,22]. This concept has been used in many branches of medical science, so it has also been taken up by the field of dentistry to analyze image patterns and medical imaging [21,22], being a valuable diagnostic tool in objectively characterizing the complexity of the structure and texture of alveolar bone [21,22,24,25,26,27,28,29,30,31,32,33].

By manipulating the grayscale of radiological images of bone trabeculae, the characteristic image of fractal analysis is obtained, making it possible to visualize the internal structure of the alveolar bone [34]. This method can be considered a reflection of the microarchitecture of trabecular bone [35,36]. Numerous studies have indicated that fractal analysis by the box-counting method can successfully evaluate trabecular changes in patients with osteoporosis [26,37,38], in patients with periodontal diseases [39], and in lactating women [40]. This analysis method is even more practical in digital dental systems, as digitizing film is no longer necessary [41]. The highest measurement accuracy of fractal analysis has been reported on cone beam computed tomography (CBCT) [12].

After reviewing the literature, we found that no study has been performed to evaluate the structure of the maxillary bones before tooth extraction and 3 months after using the fractal analysis method in the three CBCT planes. The objective of the present study was to analyze the changes produced in the alveolar bone after tooth extractions using fractal analysis on CBCT images, comparing preoperative values with postoperative values at 3 months. The null hypothesis is that the post-extraction CBCT images performed at 3 months do not differ from the initial CBCT image in the studied areas.

## 2. Materials and Methods

### 2.1. Study Design

In this retrospective study, the fractal analysis used CBCT images of patients who underwent dental extractions of maxillary and mandibular molars with natural healing of post-extraction alveoli. All data were retrieved from a database of patients from the Oral Rehabilitation Department, University of Medicine and Pharmacy in Craiova, presented for diagnosis and implant treatment between January 2021 and January 2022. Data collection was performed between January 2023 and January 2024. The study protocol was approved by the Ethics Committee of the University of Medicine and Pharmacy in Craiova (Approval Number 230/28.11.2022). Surgical interventions were undertaken with each subject’s understanding and written informed consent. Patients were treated in full compliance with applicable ethical principles, including the Helsinki Declaration of the World Medical Association (2013 version).

### 2.2. Patient Selection

The sample size was computed using G*Power 3.1.9.7, Heinrich Heine University Düsseldorf, Germany, based on the following assumptions: a significance level α of 0.05, a power 1-β equal to 0.95, and a medium effect size value (with an awareness of practical significance), resulting in a study lot of a minimum of 55 participants. Therefore, the study group consisted of 60 non-smoking, systemically healthy patients who had posterior teeth (first and/or second molars) to be extracted to plan implant treatment. The patients included in this study belonged to both sexes and were aged between 18 and 75 years. The inclusion/exclusion criteria were drawn up as follows:

Inclusion criteria:

- Patients over 18 years of age.
- Patients with first or second molars that had to be extracted.
- Patients in whom the post-extraction sockets have at least two remaining bone walls.
- Patients who do not have serious systemic diseases in ASA I or ASA II categories.
- Cooperative patients.
- Patients who want implant-prosthetic treatment.

Exclusion criteria:

- Patients with osteoporosis, severe hypertension, diabetes, kidney disease, or liver disease.
- Patients taking anticoagulants, systemic steroids, or systemic bisphosphonates.
- Patients who smoke >10 cigarettes per day, are alcohol dependent or are drug dependent.
- Pregnant or breastfeeding patients.
- Patients with a history of radiotherapy to the surgical area.
- Patients who have chosen immediate post-extraction dental implant placement.
- Patients with poor oral hygiene.

Clinical and tomographic examinations were performed on the region of interest of each patient. Each patient’s file included two CBCTs, one performed pre-extraction and one performed three months post-extraction, which were recommended to prepare the implant treatment plan.

### 2.3. Patient Evaluation

A dental chart was created for each patient, with the following steps: the clinical examination with dental record, radiological analysis, dental photographs, and blood test results. The patients accepted the individualized treatment plans developed after diagnosing each case by signing the informed consent form. After tooth extraction, the patients were recalled for follow-up at 2 weeks, 1 month, and 3 months.

### 2.4. Radiographic Evaluation

CBCT analyses were performed before and after tooth extraction using Carestream CS 8100 3D (Carestream Dental LLC., Atlanta, GA, USA), and interpretation was performed using Carestream 3D Imaging Software, 3D Suite 3.10.38.

**Figure 1:**
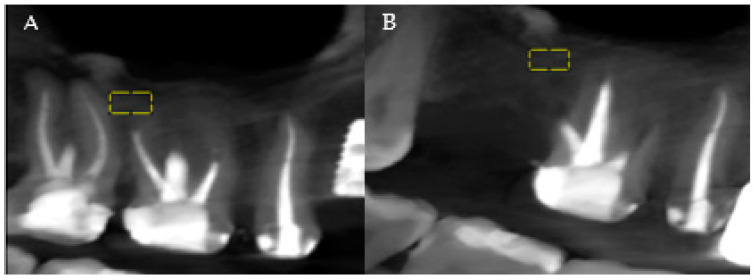
(**A**) Maxillary CBCT—before extraction. (**B**) Maxillary CBCT—after extraction. Yellow = areas of interest.

The analysis of the patients’ CBCTs from the pre-and post-extraction stages to place a dental implant was performed with both imaging software and ImageJ software (National Institutes of Health (NIH)). The precise comparison of the data obtained before and after the surgical intervention was achieved by choosing stable reference points on anatomical formations that do not change their size immediately post-extraction. At the level of the maxilla, the analysis was related to the maxillary sinus, and at the level of the mandible, the analysis was referred to the mandibular canal (Figure 1A,B, areas of interest). Measurements were performed for all three planes provided by the CBCT analysis: vertical, sagittal, and transversal.

### 2.5. Fractal Analysis

The fractal dimension measurement procedure used was the ImageJ software (version 1.54j) and the BoneJ and FracLac plugins from ImageJ. This software was chosen because it is perfect for studying trabecular bone and has the following advantages: it is free, does not require any license, and has the qualities of replicability and reproducibility.

**Figure 2:**
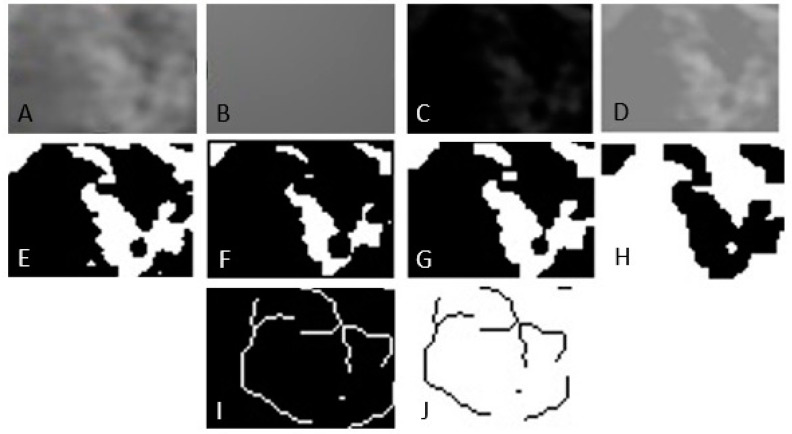
Image processing method for fractal analysis. (**A**) Duplication. (**B**) Addition of Gaussian blur filter. (**C**) Subtraction. (**D**) Addition. (**E**) Binarization. (**F**) Erosion. (**G**) Dilatation. (**H**) Inversion. (**I**) Skeletonization. (**J**) Inversion.

Print screens of the CBCT sections were selected, and the images were stored in TIFF format. A region of interest (ROI) was chosen in the lateral area adjacent to the maxillary or mandibular first or second molar, following the reference selected points on each digital image, and a fractal index was calculated for the portion of the image inside it. The ROIs were placed in the trabecular portion of the maxillary and mandibular bones. For the mandible, the ROI was limited superiorly by the portion of cortical bone and inferiorly by the mandibular canal. For the maxilla, the ROI was limited superiorly by the maxillary sinus and inferiorly by the portion of cortical bone. The same ROI was built for each digital image. The size of the molars can vary and depends mainly on the individual anatomy of each individual. In general, the roots of the upper molars are wider than the lower molars. As a result, the size of the region of interest was chosen according to the minimum mesiodistal size and the minimum height of the molar roots. The dimensions and shapes of the rectangle ROIs were 40 × 20 pixels. They were selected in such a way as to avoid overlapping anatomical formations in the area, such as roots, mandibular canal, and maxillary sinus. The sequence of steps to calculate the fractal dimension was the following (Figure 2):

1. Selecting the region of interest (ROI).
2. Cutting and duplicating.
3. Smoothing by applying a Gaussian filter with a value of 35 to eliminate variations in image brightness.
4. Subtracting the blurred image from the original image.
5. Binarization, the trabecular bone image is set to black.
6. Erosion and dilation to reduce image noise.
7. Skeletonization of the image to be used for fractal analysis.

The fractal dimension was calculated using the box counting method.

The differences between the measurements following the fractal analysis were computed using the values before the procedure and after 3 months. Positive differences reflect bone resorption, as the measurements after 3 months are smaller than the initial ones. Negative differences reflect higher bone measurements after a recovery period of 3 months, which would indicate optimum bone regeneration and healing. Given that most values measured after the procedure were smaller than those measured before, so positive differences, most patients experienced bone resorption.

### 2.6. Statistical Analysis

Data were analyzed using SPSS (Statistical Package for Social Sciences) software, version 26 (SPSS Inc., Armonk, NY, USA). Parameters were statistically described as follows: nominal and ordinal parameters were defined as frequency distributions and associated percentages, and continuous variables were defined as the couple “mean ± standard deviation (SD)” or median values (when associated with *p*-values, for non-normally distributed values). Continuous data series were analyzed for normality using the Kolmogorov–Smirnov/Shapiro–Wilk test. Based on these results, comparisons between groups for all continuous variables were performed using the Mann–Whitney U test, and the strength and direction of the relationships between continuous series were performed using Spearman Rank Order correlation. Also, three multiple regression models were developed to determine the effects of several parameters on predicting the level of bone resorption in each CBCT plane. These models included two demographic variables (age and gender) and two clinical variables (location—maxilla/mandible—and molar type—first or second molar). *p*-values smaller than 0.05 indicate statistically significant results.

## 3. Results

### 3.1. Baseline Data

**Figure 3:**
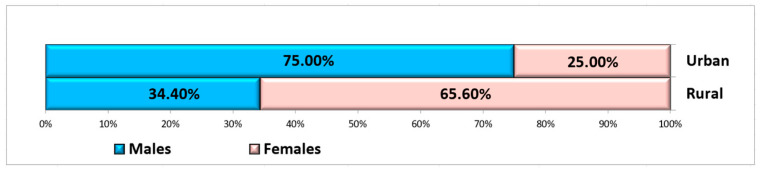
Distribution of subjects according to gender and residence area.

A total of 60 non-smoking, systemically healthy patients met the inclusion criteria to be included in this study, of whom 25 were male and 35 were female, aged between 26 and 67 years (mean: 52.29 ± 12.27 years), with a fairly even distribution between groups in terms of age and gender. The patients were selected from the Oral Rehabilitation Department of the University of Medicine and Pharmacy of Craiova, where more women than men usually present [42]. The choice of age criterion for the two groups was based on the principle of balancing the study groups. Ten patients were from rural areas, and fifty patients were from urban areas (Figure 3).

A total of 100 extractions were performed in these patients: 23 patients had only one extraction, 34 patients had two extractions, and three patients had three extractions each. Thus, 58 extractions were performed in women, and 42 extractions were performed in men. Most extractions were performed in the mandible (59%), and the majority were first molar extractions (58%) (Table 1).

The analysis of the results by age group shows that, in the maxilla, 41 extractions were performed, of which 44% were in the age group ≤ 56 years and 56% were in the age group > 56 years. In the mandible, more extractions were performed in the age group ≤ 56 years (54%), and in the age group > 56 years, there were 46%. Most extractions were of the mandibular first molar, especially in the age group ≤ 56 years, followed by the mandibular second molar and the maxillary first molar. The fewest extractions were of the maxillary second molar (Table 2).

### 3.2. Clinical Evaluation

In all patients, no complications occurred during the healing period, and post-extraction pain was of low intensity.

### 3.3. Fractal Analysis Results

Using fractal analysis on CBCT, preoperative values were compared with postoperative values at 3 months. Post-extraction bone remodeling evolution was quantified as the difference between the measurements performed before the procedure and those performed after 3 months (Table 3). The evolution was measured for all three planes, and it is defined in the following sections as vertical difference, sagittal difference, and transversal difference.

Fractal analysis showed that bone density and architecture changed after tooth extraction, and the values in the three planes were similar, with density decreasing in all three planes (Table 3).

An analysis by age revealed that the patients more frequently experienced bone remodeling, measured for all three planes, and more pronounced resorption was observed in the transversal plane (median difference 0.022 compared to 0.009 for the other two planes). A Spearman’s rank-order correlation was run to assess the relationship between the patients’ age and the evolution of vertical measurements. The preliminary analysis showed the relationship to be monotonic, as evaluated by visual inspection of a scatterplot. There was no statistically significant correlation between age and vertical difference, rs (98) = −0.145, *p* = 0.765. Similar results were obtained for sagittal and transversal differences, with no statistically significant evolutions, *p* > 0.05.

Gender analysis revealed that bone remodeling is accentuated in males compared to females, as the differences in measurements performed for all three planes are higher in males (Table 4). Overall measurements indicate that post-extraction bone remodeling is generally higher for patients with ages lower than 56 years, as well as for patients living in rural areas.

A Mann–Whitney U test was run to determine if there were differences in bone remodeling evolution between females and males for all three planes. Distributions of the differences by gender were similar, as assessed by visual inspection. Median differences for females (−0.028) and males (0.034) were statistically significantly different for sagittal measurements, U = 1548.00, z = 2.305, *p* = 0.021. Median differences were not statistically significant for vertical and transversal measurements, *p* > 0.05. Similarly, differences were not statistically significant for any of the three planes regarding the age groups or residence of the patients (Table 4).

Table 5 presents the mean values resulting from the fractal analysis, compared before and after extraction at the maxilla/mandible and at the first molar/second molar. On average, the highest values were recorded in the vertical plane at the mandible before extraction, and the lowest values were identified post-extraction in the transverse plane at the mandible. Regarding the first molar and second molar, the highest mean value was observed at the first molar post-extraction, and the lowest mean value was observed at the second molar in the transverse plane post-extraction (Table 5).

According to Table 6, maxillary/mandible measurements indicate a higher bone remodeling level for maxillary values identified in the sagittal and transversal planes and a more pronounced bone healing in the mandible for those two planes. In contrast, mandible differences in the vertical plane are higher than maxillary measurements, indicating bone remodeling in the mandible and better bone healing at the maxillary level. Statistically significant differences are identified for the vertical plane (*p* = 0.001) but not for the other two planes.

Higher bone remodeling evolutions follow extractions of the first molar compared to extraction of the second molar in both sagittal and transversal planes but not in the vertical plane, where bone healing is predominant for the first molar and bone remodeling is predominant for the second molar. Statistically significant differences are again identified for the vertical plane (*p* = 0.039) but not for the other two planes (Table 6).

Three multiple regression models were run to predict the fractal analysis evolutions for all three variables (vertical, sagittal, and transversal) from gender, age, location (maxilla/mandible), and molar type. There was linearity as assessed by partial regression plots and a plot of studentized residuals against the predicted values. There was independence of residuals, as assessed by a Durbin–Watson statistic of 1.814 for Vertical FD, 2.244 for Sagittal FD, and 1.876 for Transversal FD. There was homoscedasticity, as assessed by visual inspection of a plot of studentized residuals versus unstandardized predicted values. There was no evidence of multicollinearity, as assessed by tolerance values greater than 0.1. There were no studentized deleted residuals greater than ±3 standard deviations, no leverage values greater than 0.2, and no values for Cook’s distance above 1. The assumption of normality was met, as assessed by a Q-Q Plot.

The multiple regression model statistically significantly predicted the Vertical FD evolution, F(6,93) = 5.239, *p* < 0.0005, adj. R2 = 0.253. Three variables added statistically significantly to the prediction: age group, location, and molar type, *p* < 0.05 (Table 7).

For the Vertical FD evolution, an increase in the age group (patients with ages above 56 years) was associated with a decrease in the difference between vertical measurements (so less different values before and after).

The predicted difference (evolution) for the mandible was 0.116 greater than that predicted for the maxillary (with all values of all other independent variables being held constant). So, all other things being equal, the vertical differences in the mandible are 0.116 (on average) greater than in the maxillary. Similarly, the predicted difference (evolution) for the second molar is 0.069 greater than that predicted for the first molar (with all values of all other independent variables being held constant).

The multiple regression model did not statistically significantly predict the Sagittal FD evolution, F(6,93) = 1.150, *p* = 0.340, nor the Transversal FD evolution, F(6,93) = 1.293, *p* = 0.268.

## 4. Discussion

The detailed pre- and post-extraction bone assessment stage is of particular importance in the success of personalized implant-prosthetic treatment. Since the molar area, both maxillary and mandibular, is the most important support area in mastication and occlusion, the restoration of occlusal stops after tooth extraction can be achieved in optimal conditions only through implant-prosthetic treatment [43]. Massive bone resorption occurs post-extraction in these areas, especially in the first 3 months [44,45,46,47]. Therefore, it is essential to know the evolution of the bone during this period so that the best therapeutic decisions can be made to preserve the bone reserve (socket preservation therapies).

For this purpose, CBCT is a very useful tool for the evaluation of the three spatial planes, both of the bone dimensions and of its quality, by calculating bone density. In addition to the density assessment by CBCT (not offered by all radiological software), the fractal analysis of CBCT images brings new information regarding the state of bone mineralization and the quality of bone trabeculae. All this information is beneficial for all dentists practicing personalized implant-prosthetic therapy.

This retrospective study evaluated bone changes in the vertical, horizontal, and sagittal planes after dental extractions. By analyzing the fractal dimension on CBCT images, bone density values were compared before dental extraction and 3 months post-extraction. The same study period was chosen in other studies in the literature [44,45,46,47].

Only molars one and two were included in this study to homogenize the studied groups and to minimize, as much as possible, the structural differences of the alveolar bone. Significant post-extraction resorption occurs at the molar level, especially in the vertical dimension of the alveolar process [2].

The same protocol was used for all patients, and no complications were reported during the healing period; however, most patients experienced a reduction in bone volume. Most patients were female, and the majority of extractions were of mandibular molars.

In recent years, implant treatment has become the first choice for edentulous prosthetics. An accurate preoperative evaluation is crucial for planning dental implant therapy [48,49,50,51,52,53]. The quality of the bone structure is defined by the degree of mineralization, bone morphology, physiology, and the type of trabecular pattern [54,55,56,57,58]. Understanding the structure and microarchitecture of the jaw bones helps choose an ideal therapeutic intervention method [57]. Bone mineral density (BMD) is one of the parameters that most strongly indicates bone quality [56,59,60,61]. It is essential to prepare the bone for implant treatment by controlling the resorption and remodeling phenomena that occur during the healing process [44,45,46].

Numerous studies have demonstrated a significantly greater decrease in bone thickness in the crestal third of the alveolar bone [47,62,63]. Still, other studies have shown a significantly greater reduction in the center of the post-extraction socket [64]. Hayek et al. showed in their research that the density of the cancellous bone is directly responsible for the stability of the dental implant [65]. For this reason, the area of interest analyzed in this article was chosen not to include parts of the cortical bone but only of the trabecular bone.

Standard dental radiological analyses, such as orthopantomography, CBCT, bitewing radiographs, and cephalometric analysis, have been used for fractal analysis [17,27,66]. The main advantage of CBCT is the feasibility of accurately assessing the quality of the bone structure. Other benefits of this radiological evaluation method compared to orthopantomography or conventional computer tomography are the following: a low radiation dose, minimal image distortion, higher resolution, the possibility of obtaining real-size images, and shorter acquisition times [11,13,14,27,67,68,69,70,71]. CBCT is a reliable tool for evaluating and planning implant treatment and assessing bone quality, quantity, and density [5,17,55,68,72,73,74,75,76,77,78,79,80,81,82,83,84].

Also, in recent years, numerous strategies have been developed to assess the structure of the jaw bones. Among these is fractal analysis, which has become increasingly used [85,86,87]. Fractal analysis is a precise, economical, and efficient method of describing the complexity of fractal structures [85,86,87,88,89,90]. The process of obtaining the images is essential in fractal analysis. Amuk et al.’s study demonstrated that image format can influence fractal analysis. At the same time, kVp and mA time settings do not significantly impact fractal analysis for periapical radiographs. The settings used for three-dimensional CBCT images can affect the analysis [66]. For this reason, in this study, we used CBCT images produced by the same device to eliminate this factor that creates variations in fractal analysis. The image format is essential for fractal analysis. JPEG is the most widely used format, which offers a high level of portability. However, it has been proven that the image quality is poorer than that of other formats, such as TIFF. The TIFF format is considered to be the most useful choice because it uses the LC technique [91]. In many studies in the literature in which fractal analysis was performed on images with different formats, it was found that the TIFF format had a significantly higher FD [32,66,92].

Quantification of the trabecular bone pattern uses the box-counting technique [22,88,89,93]. The increase in the fractal dimension value is directly proportional to the complexity of the bone structure [88,89].

Hayek et al. define the results of fractal analysis as bone mineral density values [65]. Reduction in bone density values corresponds to a decrease in fractal dimension [38]. Also, the same study showed that bone density had higher values in the mandible than in the maxilla [65]. A similar result was obtained in our research in the vertical plane.

Hayek et al. correlated the fractal value of intraoral radiographs in Photo Stimulable Phosphor Plate (PSP) at the molar level with the density obtained from the analysis of the bone harvested from the respective area [65]. Their study showed that a fractal value of 1.451 of the maxillary bone in the molar area corresponds to a density of 0.150 g/cm^3^, evaluated by Misch [94] as type 4. A fractal value of 1.550 in the mandibular molar area corresponds to a bone density of 0.379 g/cm^3^, representing Misch type 3 density [65,94]. Thus, they highlighted that, in the posterior maxillary area (premolar, molar), there are densities of type 3–4, with fractal values of 1.451 to 1.544, and in the posterior mandibular area, there are densities of type 2–3, with fractal values of 1.550 and 1.563 [65]. In our study, the values of the fractal analysis ranged from 0.66 to 1.378, which shows that the bone density in the lateral maxillary and mandibular areas is very low, corresponding to Misch type 4. Our result correlates with the results of another study conducted on Romanian patients, performed on CBCT, which showed that, in the lateral maxillary area, the bone density corresponded to Misch type 4, and in the lateral mandibular area, it corresponded to Misch type 3 [95]. Another study conducted on another Romanian population group showed CBCT bone density values evaluated at the level of the maxillary 1st molar in the trabecular area with an average of 557.45 HU, Misch type 4 density, and at the level of the mandibular 1st molar in the trabecular area with an average of 561.15 HU, Misch type 3 density [96].

The multiple regression model used to analyze the fractal results of bone density in the maxillary and mandibular molar areas showed correlations with age groups, jaw type, and molar type.

In general, alveolar bone is denser anteriorly and progressively decreases posteriorly. The first molar plays an essential role in mastication, taking on greater forces than the second molar [43]. Wolff’s law states that bone remodels and changes its density depending on the mechanical stress it is subjected to [97]. Because the first molar is more mechanically stressed than the second molar, the bone around it develops to withstand these greater forces, leading to greater density.

In the case of a recent extraction, there may initially be more rapid bone remodeling in the maxilla, which could explain the appearance of a higher density in the short term. In the mandible, bone resorption may be more pronounced at the cortical level, which could give the impression of an apparent decrease in local density. The mandible and maxilla are not uniform in density, as the density varies according to location, bone type, and mechanical stress [97]. The statistically significant differences observed in the measurements made using fractal analysis on CBCT in the vertical plane correlate with the important changes that occur in the vertical plane post-extraction in the two jaws. Since the comparison is made at 3 months and the bone remodeling process is not finished, which can last up to 12 months, this may represent only a transitional phase, which makes the existence of higher values post-extraction in the maxilla than in the mandible plausible. In this case, it is vital to consider the optimal moment of implant insertion to obtain the maximum result in both osseointegration and preservation of the size of the post-extraction alveolar process.

Factors that may lead to higher bone density in elderly patients are reduced bone remodeling or more prolonged exposure to mechanical stress. In young people, bone remodels more rapidly, meaning there is a balance between bone formation and resorption [98]. This process slows down in older people, and cortical bone can become denser but also more fragile (bone sclerosis) [99]. Older patients have been subjected to mechanical stress for longer, which can stimulate osteogenesis and increase bone density in stress areas. Although bone can be dense, it can also be less vascular and more fragile [100].

The decision to extract a tooth, especially a molar, should be accompanied by a decision regarding a socket preservation modality or immediate implantation [2]. Only in this way can the situation be avoided in which extensive significant resorption has already occurred, and the solutions available for performing implant-prosthetic treatment in good conditions involve either avoiding the molar area (as in all on x or fast and fix solutions) [101] or complex interventions with lower chances of success (such as a sinus lift) [102]. All this information is even more important as patients age [103].

The limitations of this study are that it was conducted at a single university, being mono-centric, and the sample may not represent broader demographic variations.

## 5. Conclusions

Fractal analysis of CBCT in the three planes showed that the bone density of the maxillary and mandibular molar trabecular area was low pre- and post-extraction, with values corresponding to type 4 density assessed by Misch. At 3 months post-extraction, bone density was higher in the maxilla than in the mandible and higher in the first molar than in the second molar. Age was an element that also influenced density, with individuals under 56 years having lower density post-extraction, indicating a higher regeneration rate.

All implantology specialists treating patients in this region should be aware of this data to decide based on evidence-based dentistry to improve the success rate of personalized implant-prosthetic treatment.

## Figures and Tables

**Table 1:** Demographic characteristics of patients included in the study group.

Study Variable	Category	Gender		Total
		Females (%)	Males (%)	
Patients	-	35	25	60 (100%)
Age (years old)	≤56	16 (53.33%)	14 (46.67%)	30 (100%)
		45.71%	56.00%	
	>56	19 (63.33%)	11 (36.67%)	30 (100%)
		54.29%	44.00%	
Residence	Rural	6 (60%)	4 (40%)	10 (100%)
		17.14%	16.00%	
	Urban	29 (58%)	21 (42%)	50 (100%)
		82.86%	84.00%	
Number of extractions	1	14 (60.87%)	9 (39.13%)	23 (100%)
		40.00%	36.00%	
	2	19 (55.88%)	15 (44.12%)	34 (100%)
		54.29%	60.00%	
	3	2 (66.67%)	1 (33.33%)	3 (100%)
		5.71%	4.00%	
No. of extracted teeth	-	58 (58%)	42 (42%)	100 (100%)
Location	Maxilla	23 (56.1%)	18 (43.9%)	41 (100%)
		39.66%	42.86%	
	Mandible	35 (59.32%)	24 (40.68%)	59 (100%)
		60.34%	57.14%	
Molar	First molar	30 (51.72%)	28 (48.28%)	58 (100%)
		51.72%	66.67%	
	Second molar	28 (66.67%)	14 (33.33%)	42 (100%)
		48.28%	33.33%	

Values emphasized in light grey represent the sum of values by column.

**Table 2:** Dental extraction characteristics in the study group.

Study Variable	Category	Age (Years Old)		Total
		≤56	>56	
Number of extractions	-	50 (50%)	50 (50%)	100 (100%)
Maxilla	Total	18 (43.90%)	23 (56.10%)	41 (100%)
	First molar	11 (45.83%)	13 (54.17%)	24 (100%)
		61.11%	56.52%	
	Second molar	7 (41.18%)	10 (58.82%)	17 (100%)
		38.89%	43.48%	
Mandible	Total	32 (54.24%)	27 (45.76%)	59 (100%)
	First molar	20 (58.82%)	14 (41.18%)	34 (100%)
		62.50%	51.85%	
	Second molar	12 (48.00%)	13 (52.00%)	25 (100%)
		37.50%	48.15%	

Values emphasized in light grey represent the sum of values by column.

**Table 3:** Average values of fractal analysis—before and 3 months after tooth extraction.

CBCT Plane	Before Tooth Extraction			After Tooth Extraction			Difference
	Min	Max	Mean ± SD	Min	Max	Mean ± SD	
Vertical plane	0.705	1.358	1.167 ± 0.117	0.748	1.354	1.154 ± 0.119	0.013
Sagittal plane	0.796	1.290	1.138 ± 0.102	0.660	1.345	1.134 ± 0.123	−0.207
Transverse plane	0.660	1.378	1.102 ± 0.162	0.660	1.312	1.074 ± 0.164	0.028

**Table 4:** Differences between the fractal analysis values recorded in the three CBCT planes before and 3 months after tooth extraction, according to median age, age groups, gender, and residence.

Study Variable	Vertical			Sagittal			Transversal		
	Median	Mean ± SD	*p* *	Median	Mean ± SD	*p* *	Median	Mean ± SD	*p* *
Age (median)	0.009	0.013 ± 0.169	0.150 **	0.009	0.004 ± 0.145	0.820 **	0.022	0.027 ± 0.194	0.596 **
Age groups									
≤56 years	0.024	0.021 ± 0.186	0.611 *	0.019	−0.009 ± 0.14	0.852 *	0.025	0.051 ± 0.204	0.428 *
>56 years	0.001	0.007 ± 0.154		−0.005	0.015 ± 0.149		0.022	0.008 ± 0.185	
Gender									
Female	0.005	0.017 ± 0.178	0.914 *	−0.028	−0.019 ± 0.149	0.021 *^#^	0.022	−0.003 ± 0.19	0.232 *
Male	0.015	0.008 ± 0.157		0.034	0.035 ± 0.135		0.024	0.07 ± 0.193	
Residence									
Urban area	0.000	0.017 ± 0.171	0.597 *	0.002	0.002 ± 0.152	0.893 *	0.025	0.032 ± 0.202	0.521 *
Rural area	0.036	−0.001 ± 0.161		0.022	0.011 ± 0.109		−0.011	0.007 ± 0.154	

* Mann–Whitney U test. ** Spearman Rank Order correlation. ^#^ Statistically significant result.

**Table 5:** Average values of fractal analysis—before and after tooth extraction according to location (maxilla/mandible) and extracted tooth.

Study Variable	CBCT Plane	Before Tooth Extraction			After Tooth Extraction		
		Min	Max	Average ± SD	Min	Max	Average ± SD
Maxilla	V	0.705	1.326	1.109 ± 0.136	0.748	1.354	1.165 ± 0.112
	S	0.796	1.273	1.140 ± 0.109	0.956	1.345	1.155 ± 0.090
	T	0.660	1.378	1.159 ± 0.119	0.660	1.312	1.098 ± 0.164
Mandible	V	1.045	1.358	1.206 ± 0.081	0.759	1.333	1.145 ± 0.124
	S	0.799	1.29	1.136 ± 0.098	0.660	1.325	1.119 ± 0.141
	T	0.660	1.378	1.062 ± 0.177	0.660	1.249	1.058 ± 0.163
First Molar	V	0.705	1.358	1.170 ± 0.108	0.937	1.354	1.186 ± 0.096
	S	0.796	1.273	1.125 ± 0.119	0.660	1.321	1.125 ± 0.136
	T	0.660	1.378	1.106 ± 0.176	0.660	1.312	1.082 ± 0.166
Second Molar	V	0.796	1.326	1.161 ± 0.128	0.748	1.292	1.107 ± 0.133
	S	0.991	1.290	1.155 ± 0.07	0.913	1.345	1.146 ± 0.103
	T	0.719	1.314	1.096 ± 0.143	0.660	1.265	1.064 ± 0.162

V—vertical plane, S—sagittal plane, T—transversal plane.

**Table 6:** Differences between the fractal analysis values recorded in the three CBCT planes before and 3 months after tooth extraction, according to location (maxilla/mandible) and extracted tooth.

Study Variable	Vertical			Sagittal			Transversal		
	Median	Mean ± SD	*p* *	Median	Mean ± SD	*p* *	Median	Mean ± SD	*p* *
Location									
Maxilla	−0.034	−0.056 ± 0.177	0.001 ^#^	0.023	−0.015 ± 0.147	0.790	0.050	0.061 ± 0.153	0.129
Mandible	0.034	0.062 ± 0.145		−0.013	0.017 ± 0.144		−0.013	0.004 ± 0.216	
Molar									
First Molar	−0.004	−0.016 ± 0.145	0.039 ^#^	0.015	0 ± 0.153	0.955	0.025	0.024 ± 0.202	0.867
Second Molar	0.055	0.054 ± 0.191		−0.022	0.009 ± 0.135		0.019	0.032 ± 0.185	

* Mann–Whitney U test. ^#^ Statistically significant result.

**Table 7:** Multiple regression model: Linear coefficients—Vertical differences.

Parameter	B	t	Sig	CI interval	
				Lower	Upper
Gender	0.027	−1.188	0.238	−0.381	0.096
Age group	0.159	−3.286	0.001 ^#^	−0.013	−0.003
Maxilla/mandible	0.116	2.603	0.011 ^#^	0.038	0.280
Molar (First/Second Molar)	0.069	3.753	0.000 ^#^	0.055	0.177

^#^ Statistically significant values.

## Data Availability

The authors declare that the data of this research are available from the corresponding authors upon reasonable request.

## Abbreviations

The following abbreviations are used in this manuscript:

CBCT	Cone Beam Computed Tomography
FD	Fractal Dimension
ASA	American Society of Anesthesiologists
NIH	National Institutes of Health
ROI	Rectangular Region of Interest
BMD	Bone Mineral Density
PSP	Photo Stimulable Phosphor Plate
HU	Hounsfield Units
